# Dose-Dependent Porcine Deltacoronavirus Infection Reveals Linkage Between Infectious Dose and Immune Response

**DOI:** 10.3390/ani15172536

**Published:** 2025-08-28

**Authors:** Xiaocheng Bao, Liangxin Xia, Wenbin Bao, Ming’an Sun, Shuai Zhang

**Affiliations:** 1 College of Veterinary Medicine, Yangzhou University, Yangzhou 225009, China; mx120240996@stu.yzu.edu.cn (X.B.); mingansun@yzu.edu.cn (M.S.); 2 College of Animal Science and Technology, Yangzhou University, Yangzhou 225009, China; lxxia0830@163.com (L.X.); wbbao@yzu.edu.cn (W.B.)

**Keywords:** PDCoV, infection dose, immune response, STAT1, ISG15, MX2

## Abstract

Porcine deltacoronavirus (PDCoV), an emerging enteropathogenic coronavirus, has inflicted substantial economic losses on the global swine industry. While the severity of infectious disease depends on the dynamic interplay between inoculum dose and host response, the molecular mechanism by which PDCoV dose modulates host immunity remains unclear. Hence, we systematically compared the transcriptomic changes in intestinal epithelial cells infected with different doses of PDCoV, and investigated the relationships between inoculum dose, host immune responses, and disease progression. PDCoV replication peaked at 24 h post-infection, and host responses showed a distinct dose-dependent pattern, with high-dose infection inducing more extensive transcriptional remodeling than low-dose infection. Both doses significantly activated the transcription of STAT1 and its downstream interferon-stimulated genes, while high-dose infection additionally triggered a cytokine storm characterized by excessive IL-6 and TNF-α expression. Functional validation demonstrated that STAT1 overexpression markedly inhibited PDCoV infection by enhancing ISRE promoter activity, and overexpression of its downstream ISG15 and MX2 also exerted independent and significant antiviral effects. These findings reveal the biphasic nature of PDCoV dose-dependent regulation of immunopathological mechanisms and identify STAT1 and specific ISGs (ISG15, MX2) as potent antiviral effectors, providing crucial insights into PDCoV pathogenicity and offering promising targets for developing immunomodulatory therapeutics or vaccines to control PDCoV outbreaks in swine.

## Simple Summary

Porcine deltacoronavirus (PDCoV) is an emerging intestinal virus causing heavy losses in global swine farming. Its infection severity is linked to viral dose and host response, but the underlying mechanisms were unclear. Our study compared porcine intestinal epithelial cell responses to different PDCoV doses. We found that the virus replicates most actively 24 h post-infection, with host reactions showing clear dose dependence; high doses induce more extensive gene activity changes. Both doses activate the immune molecule STAT1 and its downstream antiviral genes, but high doses also trigger excessive inflammation via IL-6 and TNF-α. We confirmed that boosting STAT1, ISG15, or MX2 levels strongly inhibits PDCoV. These findings advance our understanding of the interplay between initial doses and host responses, providing mechanistic insights into PDCoV pathogenesis.

## 1. Introduction

Porcine deltacoronavirus (PDCoV), an emerging coronavirus, poses a significant threat to the swine industry. First detected in porcine fecal samples in Asia, it was not recognized as an etiologic agent until 2014 [1,2]. Subsequently, PDCoV infections have been reported in multiple countries, including Canada, China, South Korea, Thailand, Laos, Vietnam, and Japan [3,4,5,6]. PDCoV, like other enteric coronaviruses such as porcine epidemic diarrhea virus (PEDV) and transmissible gastroenteritis virus (TGEV), primarily infects intestinal epithelial cells, inducing diarrhea, vomiting, and dehydration [7]. PDCoV is highly contagious, primarily transmitted via the fecal–oral route. Airborne transmission has also been reported, likely occurring through ingestion of aerosolized virus particles [8]. The clinical progression of PDCoV infection shows distinct age-dependent severity. Neonatal piglets, particularly those born from seronegative dams, exhibit significantly more severe symptoms, often progressing to lethality. In contrast, older swine (e.g., weaned pigs and adult sows) demonstrate high infection rates but substantially lower mortality risk [9]. Notably, emerging evidence indicates that PDCoV exhibits cross-species transmission potential, with confirmed infections documented in mice, turkeys, calves, and even humans [10,11,12,13], emphasizing the importance of research dedicated to its pathogenesis mechanisms. Meanwhile, given the substantial economic losses inflicted by PDCoV on the swine industry, it is crucial to thoroughly dissect the mechanisms by which the virus manipulates host immune responses. However, numerous mysteries remain regarding PDCoV’s pathogenic mechanisms and its interplay with the host’s innate immunity.

Interferon (IFN) acts as the core defense for cells against viral infections. Upon viral infection, type I interferons bind to a heterodimeric transmembrane receptor composed of IFNAR1 and IFNAR2 subunits, thereby activating the Janus kinase (JAK)–signal transducer and activator of transcription (STAT) pathway. In this canonical signaling cascade, activated JAK1 and tyrosine kinase 2 (TYK2) phosphorylate cytosolic STAT1 and STAT2, triggering their dimerization, nuclear translocation, and subsequent binding to interferon regulatory factor 9 (IRF9) to form the trimeric interferon-stimulated gene factor 3 (ISGF3) complex. This complex then binds to interferon-stimulated response elements (ISREs) upstream of interferon-stimulated genes (ISGs), initiating their transcription and ultimately establishing an antiviral state within the cell [14]. Notably, the relationship between inoculum dose (defined as the initial number of pathogens during infection), viral invasion ability, and host immune responses has not fully elucidated. Elucidating this dose–response relationship is pivotal for deciphering how infectious dose modulates immune responses, and, consequently, disease progression. Accumulating experimental and clinical studies on various pathogens have highlighted the significance of initial inoculum dose in shaping host responses and disease outcomes. Data from human volunteer challenge studies involving various influenza strains consistently demonstrate that symptom severity escalates in a viral dose-dependent manner [15,16]. Additionally, in SARS patients, higher nasopharyngeal viral loads were associated with significantly increased disease severity and viral shedding [17]. Similarly, human volunteer challenge studies have established a dose–response relationship between human coronavirus 229E and infection severity [18]. Despite these advances, research on the dose–response relationship in PDCoV, especially the association between initial infection dose and host immune responses, remains limited.

Therefore, this study aimed to decipher the molecular response mechanisms of porcine intestinal epithelial cells to different PDCoV inoculum doses and systematically analyze the changes in viral gene expression and host transcriptome using RNA sequencing technology. We found that high-dose PDCoV infection, compared to low-dose infection, elicits exaggerated host immune responses characterized by significantly elevated expression levels of cytokines and IFN-related genes. Further studies confirmed that PDCoV infection could significantly upregulate the expression of STAT1, IFN-β, and their downstream ISGs such as ISG15, MX1, MX2, and OAS1. Overexpression of STAT1 could significantly inhibit the transcription and protein of PDCoV N gene by activating the activity of ISRE promoter, thereby restricting viral replication. Additionally, individual overexpression of ISG15 or MX2 could also independently exert antiviral effects, significantly reducing the intracellular PDCoV load. These findings not only provide a new perspective for an in-depth understanding of the pathogenic mechanism of PDCoV (especially the dose-dependent regulatory law of host immunity) but also lay a theoretical foundation for the development of antiviral strategies targeting the STAT1-ISGs axis.

## 2. Materials and Methods

### 2.1. Cells and Virus

IPEC-J2 cells (provided by the University of Pennsylvania, Philadelphia, PA, USA) and ST cells (ATCC, Manassas, VA, USA) were preserved in our laboratory. Both cell lines were maintained in Dulbecco’s modified Eagle’s medium (DMEM, Gibco, Gaithersburg, MD, USA) supplemented with 10% fetal bovine serum (FBS), 100 U/mL penicillin, and 100 μg/mL streptomycin, under conditions of a 37 °C, 5% CO_2_, humidified atmosphere. Cells were seeded at 2 × 10^5^/mL in 25 cm^2^ plastic tissue culture flasks and passaged every 3–4 days, with a maximum of 30 passages.

The PDCoV CHN-GD16-05 strain was kindly provided by Professor Zhenhai Chen from the College of Veterinary Medicine, Yangzhou University.

### 2.2. Reagents and Antibodies

Fetal bovine serum (FBS) was obtained from Nanjing BioChannel Biotechnology Co., Ltd. (Nanjing, China). Penicillin–streptomycin liquid and RIPA lysis buffer were obtained from Solarbio (Beijing, China). The BCA Protein Quantification Kit, SuperPico ECL Chemiluminescence Kit, HiScript Q RT SuperMix for qPCR (+gDNA wiper), and ClonExpress Ultra One Step Cloning Kit were purchased from Vazyme (Nanjing, China). SuperStar Universal SYBR Master Mix was obtained from CWBIO (CW3360M, Taizhou, China). RNAiso Plus was purchased from TaKaRa (Dalian, China). Millipore PVDF membranes were purchased from Merck Chemical Technology Co., Ltd. (Shanghai, China). Poly (I:C) was purchased from APExBIO (Boston, MA, USA). X-tremeGENE HP DNA Transfection Reagent was from Roche (Basel, Switzerland). The Dual Luciferase Reporter Gene Assay Kit was obtained from Yeasen Biotechnology (Shanghai, China).

Mouse anti-PDCoV-N monoclonal antibody (IF, 1:200; Western blotting, 1:1000) was purchased from Medgene Labs (Brookings, SD, USA). DyLight 488 goat anti-mouse IgG (H+L) was purchased from Abbkine Scientific Co., Ltd. (Wuhan, China). 4′,6-diamidino-2-phenylindole (DAPI) was obtained from Life Technologies (Carlsbad, CA, USA). Rabbit anti-STAT1 antibody was obtained from Bioworld Technology (Louis Park, MN, USA). HRP-conjugated rabbit anti-GAPDH was obtained from ABclonal Technology (Wuhan, China). Rabbit anti-ISG15 antibody, HRP-conjugated goat anti-mouse IgG (H+L), and goat anti-rabbit IgG (H+L) were purchased from Proteintech (Wuhan, China).

### 2.3. Plasmid Construction

pRL-TK plasmid, IFN-stimulated response elements (ISREs) luciferase reporter plasmid, and pCDNA3.1-HA plasmid were resident in our laboratory. STAT1 and ISG15 were cloned into pCDNA3.1-HA (using KpnI/BamHI), resulting in pCDNA3.1-HA-STAT1 and pCDNA3.1-HA-ISG15.

### 2.4. Virus Infection

IPEC-J2 cells at 80% confluency were infected with PDCoV at 37 °C for 1 h. The inoculum was then removed, and cells were rinsed to eliminate unattached virus before being incubated in DMEM containing 10 μg/mL trypsin for specified durations at 37 °C. Infection was assessed via RT-qPCR, Western blotting, and immunofluorescence.

### 2.5. RNA Extraction and Quantitative Real-Time PCR (RT-qPCR)

Total RNA was extracted using RNAiso Plus per the manufacturer’s protocol. RNA quality and concentration were assessed via agarose gel electrophoresis and Nanodrop, respectively. One microgram of total RNA was reverse-transcribed into cDNA using HiScript Q RT SuperMix, followed by qPCR with SuperStar Universal SYBR Master Mix on an Applied Biosystems 7500 Fast Real-Time PCR System. *GAPDH* served as the internal reference, and the relative transcript levels of target genes were calculated using the 2^−ΔΔCt^ method across three biological replicates. Primers were designed using primer-BLAST (https://www.ncbi.nlm.nih.gov/tools/primer-blast/index.cgi (accessed on 16 September 2024)) and verified via melt curve analysis for efficiency. Primer sequences are listed in Table S1.

### 2.6. Western Blotting

Cells were lysed in RIPA buffer with 1:100 protease inhibitor cocktail on ice for 30 min, and centrifuged (12,000× *g*, 15 min, 4 °C) to collect the supernatant. Total protein concentration was measured via a BCA Protein Quantification Kit. A measure of 50 μg of protein per sample with 5× loading buffer was separated on 10% SDS-PAGE gels, then transferred to Millipore PVDF membranes activated by methanol (30 s). Membranes were then blocked with 5% nonfat milk in TBST (0.1% Tween 20 in Tris-buffered saline) and incubated overnight at 4 °C with primary antibodies (1:1000). After three TBST washes, membranes were incubated with HRP-conjugated goat anti-mouse/rabbit secondary antibodies (1:5000) for 1 h, rewashed, and visualized using ECL substrate (1:1 mix of A/B) with a Tianneng imaging system. Bands were quantified via Quantity One software v4.62 (Bio-Rad, Hercules, CA, USA) and normalized to GAPDH.

### 2.7. Plaque Assay

Serial ten-fold viral dilutions in DMEM/trypsin (10 μg/mL) were added to confluent ST cell monolayers in 12-well plates and incubated at 37 °C for 2 h. After three PBS washes, cells were overlaid with 0.7% low-melting-point agarose in DMEM/trypsin and incubated at 37 °C for 72 h. Plaques were visualized by staining with 1% crystal violet in methanol.

### 2.8. Indirect Immunofluorescence

IPEC-J2 cells were seeded on coverslips in 24-well plates, infected with PDCoV for specified durations at 37 °C, then fixed in 4% paraformaldehyde for 20 min at RT. After permeabilization with 0.1% Triton X-100 in PBS for 5 min, they were rinsed and blocked with 5% bovine serum albumin for 1 h. Cells were incubated with primary antibody (1:100) overnight at 4 °C. Following PBS washes, samples were incubated with secondary antibody (1:200) for 30 min at RT, rewashed three times with PBS, and stained with 1 μg/mL DAPI for 10 min. Images were captured using a Leica TCS SP8 STED laser scanning confocal microscope (Leica, Mannheim, Germany) and analyzed using Leica LAS AF Lite (Leica).

### 2.9. Transcriptome Sequencing Analysis

IPEC-J2 cells were infected with L_PDCoV (MOI = 0.1) or H_PDCoV (MOI = 10), and mock-infected with DMEM, and harvested after 24 h for RNA extraction. RNA quality was assessed via Agilent 5400 (Agilent Technologies, Santa Clara, CA, USA). Novogene Co., Ltd. (Beijing, China) performed transcriptome sequencing on 1 μg of total RNA per sample. Sequencing data were mapped to *Sus scrofa* and PDCoV reference genomes. Differential expression was analyzed using the DESeq2 R package with significance cutoff of *p*-value < 0.05. Differentially expressed genes (DEGs) were identified by the screening criteria based on |log_2_(fold change)|  >  1 and adjusted *p*-value < 0.05. Volcano plots were generated using the Enhanced Volcano v1.2 R package. GO (http://geneontology.org (accessed on 16 June 2024)) and KEGG (http://www.genome.jp/kegg/ (accessed on 16 June 2024)) annotations were performed via the clusterProfiler R package (version 3.4.4); pathways with adjusted *p*-value ≤ 0.05 were considered significantly enriched. Gene set enrichment analysis (GSEA) was performed using GSEA software version 4.0.3 (http://www.broadinstitute.org/gsea (accessed on 16 June 2024)).

### 2.10. Dual Luciferase Assay

IPEC-J2 cells were co-transfected with ISRE luciferase plasmid, vector/STAT1, and pRL-TK plasmids. At 24 h post-transfection, cells were either transfected with 200 ng Poly(I:C) (positive control) or infected with PDCoV (MOI = 1), followed by a 24 h incubation. Luciferase activity in cell lysates was measured using a dual-luciferase reporter system.

### 2.11. Statistical Analysis

Data are presented as means ± standard deviation (SD) from three independent experiments. Outliers were determined by Grubbs’ test (alpha = 0.05) and excluded from the analyses. Student’s *t*-test was used to compare the significant differences between two groups and a one-way ANOVA was used for comparisons of more than two groups using GraphPad Prism 6 software. Differences were considered statistically significant at * *p*  <  0.05, ** *p*  <  0.01.

## 3. Result

### 3.1. Proliferation Kinetics of PDCoV in IPEC-J2 Cells

**Figure 1:**
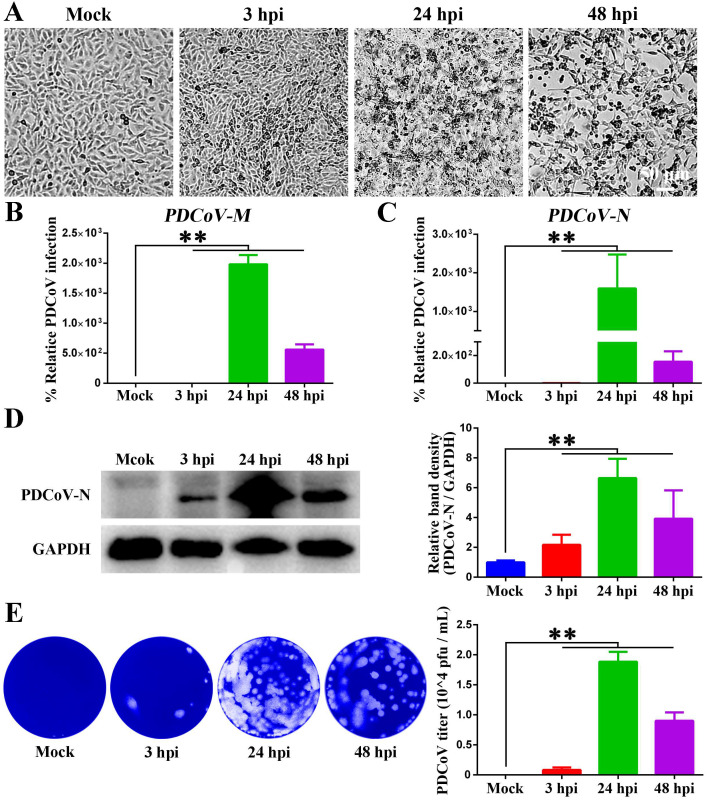
Proliferation kinetics of PDCoV in IPEC-J2 cells. (**A**) Morphological changes in IPEC-J2 cells infected with PDCoV at different time points observed by microscopy. Scale bar = 50 μm. (**B**,**C**) RT-qPCR analysis of PDCoV-M (**B**) and PDCoV-N (**C**) mRNA levels in IPEC-J2 cells at different time points post-PDCoV infection. (**D**) Western blotting analysis of PDCoV-N protein expression in IPEC-J2 cells at different time points post-infection. (**E**) Culture supernatants from (**D**) were titered by plaque assay in ST cells. ** *p* < 0.01.

IPEC-J2 cells were incubated with PDCoV at a multiplicity of infection (MOI) of 1 to determine viral replication kinetics. The cytopathic effects (CPEs) were monitored for 48 h, and progressive CPEs exacerbation in PDCoV-infected cells. No significant gross lesions were observed in the 3 h group compared to the mock group. Distinct CPEs were visible at 24 h post-infection (hpi), and PDCoV caused significant host cell rounding, necrotic cell death, and monolayer detachment at 48 hpi (Figure 1A). The intracellular *PDCoV-M* and *N* mRNA levels, PDCoV-N protein levels, and extracellular viral titers were quantified by RT-qPCR, Western blotting, and plaque assay, respectively. RT-qPCR results demonstrated that the *PDCoV-M* and *N* mRNA levels increased steadily over time and reached their peak at 24 hpi (Figure 1B,C). Additionally, the Western blotting result showed that PDCoV-N protein expression increased with time, reaching a maximal level at 24 hpi, followed by a decline at 48 hpi (Figure 1D). Meanwhile, supernatants were harvested at the corresponding time points for virus titration by plaque assay in ST cells, and viral titers paralleled the peak of viral mRNA and protein expression at 24 hpi (Figure 1E). These findings showed that IPEC-J2 is a permissive cell line for PDCoV infection, with viral infection peaking at 24 hpi.

### 3.2. The Outcome of PDCoV Infection Depends on the Dose of Infection

**Figure 2:**
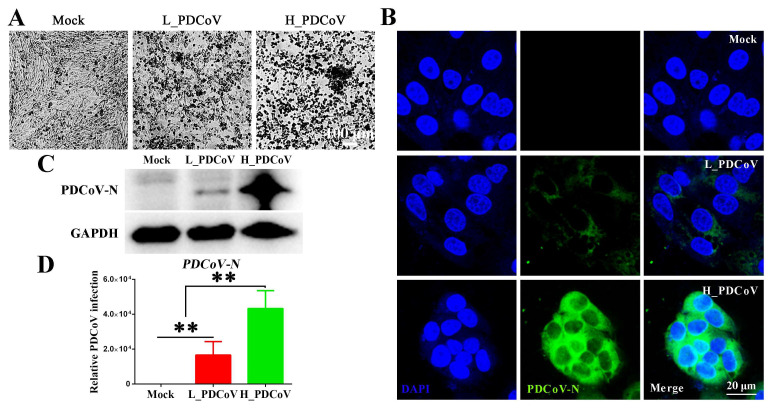
The outcome of PDCoV infection depends on the dose of infection. (**A**) Morphological changes in IPEC-J2 cells infected with different dose of PDCoV at 24 hpi observed by microscopy. Scale bar = 100 μm. (**B**) IPEC-J2 cells were infected with L_PDCoV (MOI = 0.1) or H_PDCoV (MOI = 10) and fixed at 24 hpi. Cells were then stained for confocal microscopy using mouse anti-PDCoV-N mAb, followed by DyLight 488-conjugated goat anti-mouse IgG (green). Nuclei were stained with DAPI (blue). Scale bar = 20 μm. (**C**) Western blotting analysis of PDCoV-N protein expression in IPEC-J2 cells at 24 hpi. (**D**) RT-qPCR analysis of PDCoV-N mRNA levels in IPEC-J2 cells 24 hpi. ** *p* < 0.01.

To evaluate the relationship between viral inoculum dose and peak viral load, IPEC-J2 cells were infected with PDCoV at low (MOI = 0.1) and high (MOI = 10) doses. As shown in Figure 2A, the high-dose PDCoV-inoculated group (H_PDCoV) elicited markedly exacerbated CPEs (cell rounding and monolayer detachment) compared to low-dose challenges (L_PDCoV) at 24 hpi. In addition, IFA revealed significantly higher positive signals in high-dose infections than low-dose infections (Figure 2B). Consistent with these findings, Western blotting and RT-qPCR analysis also revealed higher PDCoV-N expression in the high-dose group than in low-dose group (Figure 2C,D). These results established a link between inoculum dose and cytopathic effects, PDCoV can be detected both in low- and high-dose infection groups, and high-dose PDCoV infection exhibited robust viral protein expression, which may contribute to accelerated cytopathology and severe clinical disease occurrence.

### 3.3. Transcriptional Profiles of Differently Dosed PDCoV-Infected IPEC-J2 Cells

**Figure 3:**
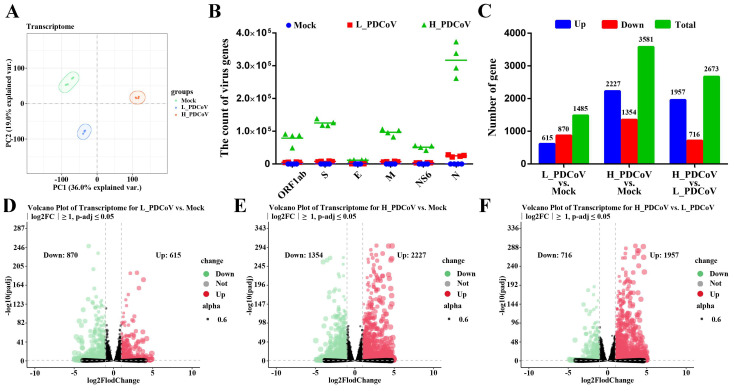
Transcriptional profiles of differently dosed PDCoV-infected IPEC-J2 cells. (**A**) Principal component analysis (PCA) plot of principal component 1 (PC1) and principal component 2 (PC2). PC1 explained 19.0% of the variation while PC2 represented 36.0%. (**B**) Viral gene transcript levels of mock-, L_PDCoV (MOI = 0.1)-, and H_PDCoV (MOI = 10)-infected IPEC-J2 cells at 24 hpi were measured by RNA-seq. The transcript levels of viral genes are shown as count values. (**C**) Differential gene expression of L_PDCoV vs. mock, H_PDCoV vs. mock, and H_PDCoV vs. L_PDCoV were assessed by RNA-seq. (**D**–**F**) Volcano plots were used to visualize statistically significant expression changes (|log2FC| > 1 and adjusted *p*-value < 0.05) for each comparison group, with the number of DEGs shown on the left and right.

To unveil the relationship between viral infectious dose and host responses after PDCoV infection, mock and IPEC-J2 cells infected with different infectious doses of PDCoV were collected at 24 hpi for transcriptome sequencing. Principal component analysis (PCA) showed a clear separation between the three groups and a high similarity among the four biological replicates within each treatment (Figure 3A). Viral genomic RNA (*ORF1a*, *S*, *E*, *M*, *NS6*, and *N*) were detected in all infected groups, and significantly higher copy numbers were found in high-dose cohorts compared to low-dose counterparts (Figure 3B). Furthermore, differential expression analysis (|log2FC| ≥ 1, *p*-adj ≤ 0.05) was performed to identify the transcriptomic differences among groups. Compared to mock-infected cells, the transcriptome data revealed 5066 DEGs in total, and both low and high-dose PDCoV-infected groups exhibited extensive transcriptional reprogramming. As expected, the number of DEGs were more pronounced in cells inoculated with high than that with low doses. The low-dose PDCoV-infected group showed 870 downregulated genes and 615 upregulated genes, while the high-dose PDCoV-infected group had 1354 downregulated genes and 2227 upregulated genes. Meanwhile, direct comparison between high- and low-dose PDCoV-infected groups revealed 716 downregulated and 1957 upregulated genes (Figure 3C). Volcano plots further visualized these dose-specific expression profiles (Figure 3D,F). To further validate the accuracy of the transcriptomic data, we randomly selected five upregulated DEGs (including *IL6*, *ISG20*, *MX2*, *TNF-α*, and *TRIM21*) and six downregulated DEGs (including *C3*, *CHI3L1*, *F3*, *MEGF9*, *SOD3*, and *TIMP2*) for RT-qPCR analysis. Consistent with the RNA sequencing results, the mRNA levels of these upregulated DEGs were significantly increased, while those of the downregulated DEGs were significantly decreased, indicating the reliability of the RNA sequencing data (Figure S1). These data collectively suggest a significant link between infectious dose and host transcriptional responses, and that the outcome of PDCoV infection in IPEC-J2 cells varies with the different infectious virus doses.

### 3.4. The Dose-Dependent Effects of PDCoV Infection on Host Gene Function and Signaling Pathways

**Figure 4:**
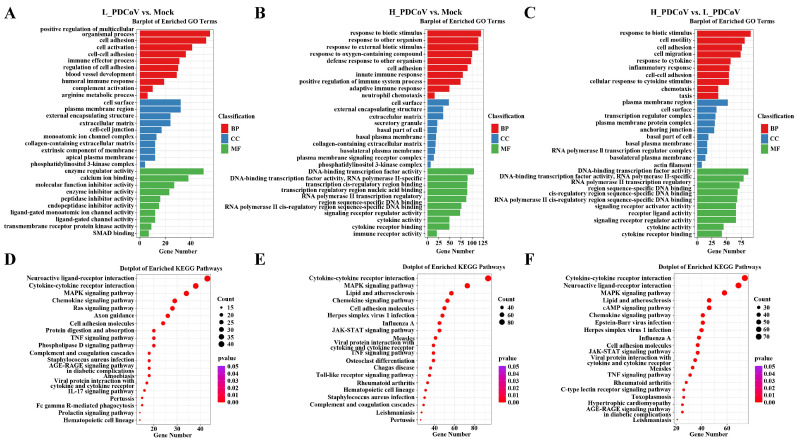
The dose-dependent effects of PDCoV infection on host gene function and signaling pathways. (**A**) GO analysis of DEGs between the L_PDCoV and mock groups. (**B**) GO analysis of DEGs between the H_PDCoV and mock groups. (**C**) GO analysis of DEGs between the H_PDCoV and L_PDCoV groups. (**D**–**F**) KEGG analysis of DEGs between the L_PDCoV and mock, H_PDCoV and mock, and H_PDCoV and L_PDCoV groups.

All DEGs were annotated to the Gene Ontology (GO) database (http://geneontology.org (accessed on 16 June 2024), taking corrected qvalue ≤ 0.05 as a threshold), and enriched to 150 signaling pathways in L_PDCoV vs. Mock, 209 signaling pathways in H_PDCoV vs. Mock, and 240 signaling pathways in H_PDCoV vs. L_PDCoV, respectively (Table S2). Comparatively, high-dose PDCoV infection significantly enriched more GO terms related to biological processes (BP), cellular components (CC), and molecular functions (MF) compared to low-dose infection. Intriguingly, high-dose PDCoV infection induced a significantly broader range of transcriptional responses, including cytokine-related pathways (e.g., cytokine production, regulation of cytokine production, cellular response to cytokine stimulus, response to cytokine, cytokine-mediated signaling pathway, and cytokine activity) and immune response pathways (e.g., defense response to virus, response to virus, innate immune response, regulation of immune response, regulation of immune system process, antiviral innate immune response, and activation of immune response) (Figure 4A,C).

The KEGG pathway enrichment analysis (http://www.genome.jp/kegg/ (accessed on 16 June 2024)) reveals that both low-dose PDCoV and high-dose PDCoV infections significantly activate immune-related pathways, such as cytokine–cytokine receptor interaction and MAPK signaling pathway (Figure 4D,F). Notably, high-dose PDCoV infection shows a stronger activation of these pathways compared to low-dose PDCoV infection, indicating a dose-dependent effect. Compared with the mock group, 94 DEGs in the high-dose PDCoV infection group were annotated to cytokine–cytokine receptor interaction, whereas only 38 DEGs in the low-dose PDCoV infection group showed similar enrichment (Table S3). This dose-dependent hyperactivation of cytokines aligned with the CPE observation. These results collectively suggest that PDCoV infection can significantly activate the host’s immune and inflammatory responses, regulate host cell signal transduction and transcriptional processes, and show that its biological effects exhibit dose-dependent characteristics. These dose-dependent transcriptional dynamics, particularly the hyperactivation of cytokine pathways at high viral inoculum, may mirror critical events in porcine intestinal infection, where rapid viral replication kinetics could potentially overwhelm host innate immune defenses within 24 h, thereby influencing clinical outcomes.

### 3.5. Assessment and Real-Time qPCR Verification of Genes Related to Defense Response to Virus

A detailed GO enrichment analysis of upregulated DEGs revealed that both L_PDCoV and H_PDCoV infections induce significant antiviral defense responses, with H_PDCoV triggering a more pronounced effect than L_PDCoV. Notably, the consistent enrichment of “defense response to virus” across all comparisons highlights the critical role of this biological process in the host’s response to PDCoV infection (Figure S2A–C). Additionally, a Venn diagram was generated to examine the overlap of DEGs across different comparison groups (L_PDCoV vs. mock, H_PDCoV vs. mock, and H_PDCoV vs. L_PDCoV), with a red circle highlighting 247 genes differentially expressed in all comparison groups. GO terms associated with these shared DEGs were also significantly enriched in the “defense response to virus” pathway (Figure S2D).

**Figure 5:**
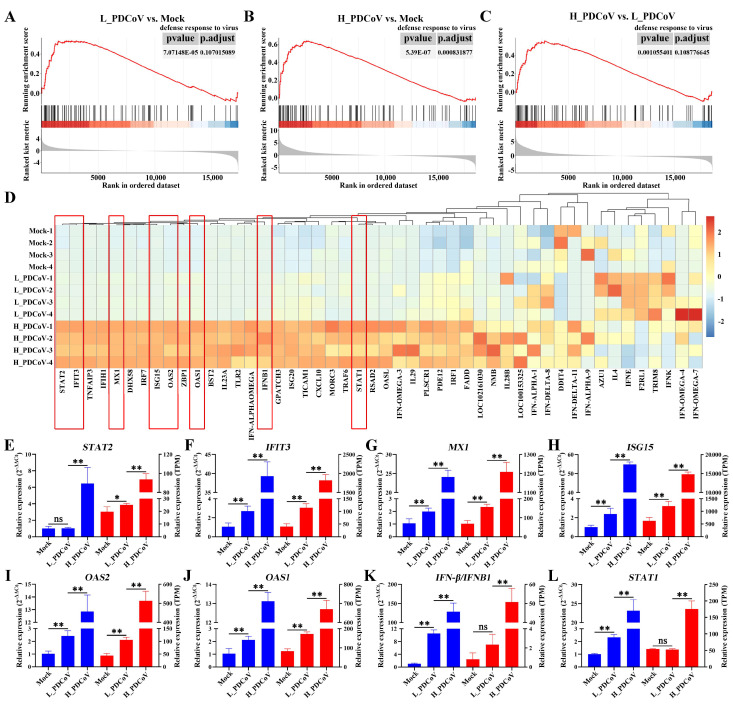
Assessment and real-time qPCR verification of genes related to defense response to virus. (**A**–**C**) Gene set enrichment analysis (GSEA) plots of defense response to virus-related genes in comparisons between the L_PDCoV and mock groups (**A**), the H_PDCoV and mock groups (**B**), and the H_PDCoV and L_PDCoV groups (**C**). (**D**) Heat map of defense response to virus-related genes in different samples (mock, L_PDCoV, H_PDCoV). The red boxs indicate genes related to “defense response to virus”, which were also strongly altered. (**E**–**L**) The mRNA levels of *STAT2* (**E**), *IFIT3* (**F**), *MX1* (**G**), *ISG15* (**H**), *OAS2* (**I**), *OAS1* (**J**), *IFN-β/IFNB1* (**K**), and *STAT1* (**L**) were compared with RT-qPCR. Gene expression levels, as measured by RT-qPCR, are plotted on the left axis, while RNA-seq expression levels in TPM units are plotted on the right axis. ns: not significant; * *p* < 0.05; ** *p* < 0.01.

GSEA showed that both L_PDCoV and H_PDCoV infections significantly affected the expression of genes related to “defense response to virus” compared with the mock group, indicating that PDCoV infection can significantly alter the host’s immune response (Figure 5A,B). Figure 5C showed that there are also significant differences between high-dose and low-dose PDCoV infections, suggesting that viral dose may affect the intensity of the host’s immune response. Furthermore, the transcriptional profile of genes related to “defense response to virus” further corroborate this finding, with high-dose infections inducing a more robust immune response in the host (Figure 5D and Table S4). The expression pattern of genes related to “defense response to virus” were also strongly altered. It is noteworthy that, compared with L_PDCoV infection, H_PDCoV infection significantly enhanced the expression of type I interferon (IFN-β) and activated the downstream IFN signaling pathway. Meanwhile, high-dose infection significantly upregulated the expression levels of *STAT1* and *STAT2*, which further induced the expression of multiple ISGs such as *IFIT3*, *MX1*, *ISG15*, *OAS2*, and *OAS1* (Figure 5E–L).

### 3.6. Interactions of STAT1, ISG15, and MX2 with PDCoV in Antiviral Responses

**Figure 6:**
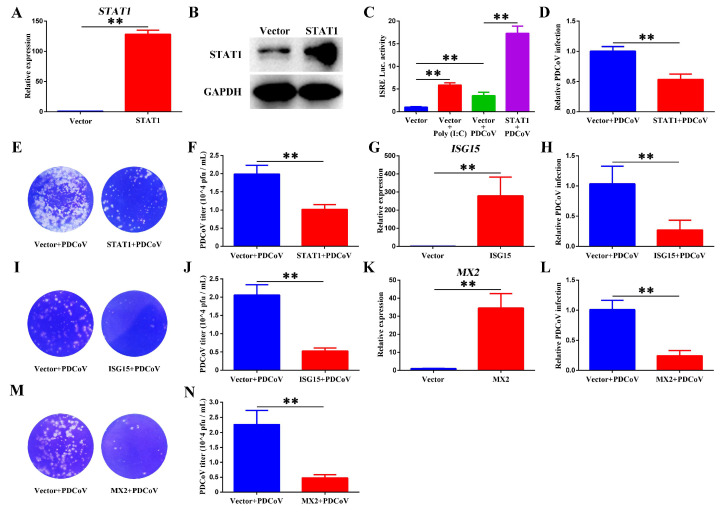
Interactions of STAT1, ISG15, and MX2 with PDCoV in antiviral responses. (**A**) RT-qPCR analysis of STAT1 mRNA levels. (**B**) Western blotting analysis of STAT1 protein expression. (**C**) The activity of ISRE-luciferase reporter gene. (**D**) PDCoV-N mRNA levels under STAT1 overexpression conditions. (**E**,**F**) Culture supernatants from (**D**) were titered by plaque assay in ST cells. (**G**) RT-qPCR analysis of *ISG15* mRNA levels. (**H**) *PDCoV-N* mRNA levels under ISG15 overexpression conditions. (**I**,**J**) Culture supernatants from Figure H were titered by plaque assay in ST cells. (**K**) RT-qPCR analysis of *MX2* mRNA levels. (**L**) *PDCoV-N* mRNA levels under MX2 overexpression conditions. (**M**,**N**) Culture supernatants from Figure L were titered by plaque assay in ST cells. ** *p* < 0.01.

STAT1 is a core transcription factor in the JAK-STAT signaling pathway, which is crucial in the host’s antiviral innate immune response. The normal functioning of STAT1 is key to the efficient initiation of type I IFN-mediated antiviral response [14]. Overexpression of STAT1 not only significantly enhances the activity of the ISRE-luciferase reporter gene but also markedly reduces PDCoV infection (Figure 6A–F), indicating the important role of STAT1 in antiviral responses. As typical ISGs downstream of ISRE, overexpression of either ISG15 or MX2 can significantly inhibit PDCoV infection (Figure 6G–N). These results demonstrate that activation of the JAK-STAT signaling pathway plays a key role in promoting the host’s defense against PDCoV infection.

## 4. Discussion

PDCoV, an emerging enteropathogenic swine coronavirus inflicting substantial economic losses on the global pig industry, primarily spreads via fecal–oral transmission through contaminated water or feed. Notably, recent detection in the respiratory tract suggests a potential respiratory transmission route [19]. This dual transmission capacity underscores the critical need to investigate interactions between viral dose, disease incidence, and clinical severity in PDCoV. Herein, we explored its dose–response relationship, finding that low-dose PDCoV infection elicits protective immune responses without significant pathology, whereas high-dose PDCoV infection overwhelms innate defenses, exacerbating disease outcomes. These results highlight the importance of dose-dependent mechanisms in shaping infection dynamics and biosecurity frameworks in modern swine production.

Infectious dose–host response data are critical for generating quantitative risk assessments of infectious agent [20]. For well-characterized viruses such as influenza A virus (IAV), MERS-CoV, SARS-CoV, and Ebola virus, prior studies have established that initial viral inoculum dose profoundly shapes host immune outcomes [21,22]. Recent research further showed that a higher dose of SARS-CoV-2 in hamsters caused more severe disease, despite no significant differences in viral load between high- and low-dose groups [23]. However, the dose–response relationship for PDCoV remains poorly defined. In this study, we investigated the link between infectious dose and host responses via transcriptomic profiling of intestinal epithelial cells, identifying dose-dependent immune signatures. We observed a distinguish host immune responses induced by different dose of PDCoV infection. Low-dose PDCoV infection triggered interferons and ISGs production, while high-dose viral inoculum not only amplified these IFN/ISG responses but also promoted pro-inflammatory cytokine release. The shift towards a pro-inflammatory cytokine environment at high PDCoV doses was similar to the cytokine storm observed in severe SARS-CoV-2. Unlike SARS-CoV-2, which primarily induces systemic cytokine storms impacting the respiratory tract and endothelium [24], PDCoV’s high-dose effect is localized to the intestinal epithelium, driving tissue-specific immunopathology. We propose that the initial PDCoV inoculum’s dose–response relationship plays a pivotal role in determining disease severity by differentially regulating host immune pathways. Specifically, low-dose viral inoculum preferentially activated type I interferon (IFN-I) signaling, initiating an ISG cascade that suppressed viral replication without inducing pathological inflammation, thereby accelerating viral clearance. Conversely, high-dose viral inoculum overwhelmed innate immune sensing thresholds, diverting signaling from protective IFN-dominated responses toward excessive inflammatory activation. This shift led to immune dysregulation, characterized by tissue injury and a permissive niche for viral persistence, ultimately exacerbating disease severity. Notably, although the time to peak viral load is consistent across different dose groups, high-dose infection continuously challenges and disrupts the host’s viral clearance barrier through inflammation-driven immune suppression and impaired antiviral synergy, highlighting the critical impact of initial inoculum dose on infection outcomes. For swine herd management, our data underscores the need to enhance surveillance of PDCoV carriers, given that even low-level viral shedding from these animals could contribute to low-dose environmental exposure, which may serve as a “stealth” source of infection in herds. For vaccination strategies, these dose-dependent infection dynamics further imply that vaccines designed to target early viral replication, particularly by boosting innate immune defenses against high-dose-induced immune suppression and reinforcing antiviral synergy, may be more effective at preventing severe clinical disease.

Innate immunity serves as the first line of defense for the host against exogenous microbial infections, relying on pattern recognition receptors (PRRs) to recognize pathogen-associated molecular patterns (PAMPs) and activate downstream signaling pathways [25]. The host triggers innate immune responses upon PDCoV infection, activating the IFN signaling pathway to initiate antiviral defense mechanisms. As a key transcription factor in the IFN response, STAT1 plays a pivotal role in regulating host antiviral immunity. STAT1 inhibits hepatitis C virus (HCV) replication by inducing IFN-λ-mediated ISGs [26]. Moreover, during PEDV infection, a swine enteric coronavirus, IPEC-J2 cells activated responses through upregulation of STAT1 and ISGs, with STAT1 overexpression significantly restricting PEDV replication [27]. In this study, both low-and high-dose PDCoV infections markedly upregulated STAT1 expression, accompanied by significant induction of downstream ISGs. We found that overexpression of STAT1 significantly enhanced ISRE promoter activity and markedly inhibited PDCoV infection, suggesting that STAT1 is a conserved host factor that inhibits the replication of porcine enterocoronavirus. Notably, downstream ISG15 and MX2 overexpression also significantly reduced PDCoV infection, demonstrating that STAT1 combats PDCoV infection by activating the transcription of ISGs. STAT1, ISG15, and MX2 are core effectors of the IFN-I pathway with established antiviral roles against multiple coronaviruses. For instance, STAT1 inhibits PEDV replication [28] and ISG15 has antiviral activity against murine hepatitis virus strain 3 [29]. However, in our study, in the high-dose infection group, elevated STAT1 and ISG expression were accompanied by a marked upregulation of pro-inflammatory cytokines, including IL-6 and TNF-α. This severely dysregulated interferon response and cytokine storm may disrupt antiviral homeostasis, potentially facilitating PDCoV persistence. The dose-dependent modulation of host responses also informs the design of more effective vaccination strategies. Vaccines which target PDCoV should therefore induce rapid, robust innate immune priming to block initial viral establishment instead of inducing dysregulated immune responses and severe tissue damage.

## 5. Conclusions

We examined transcriptional responses in intestinal epithelial cells infected with different dose of PDCoV. The analysis revealed patterns consistent with infectious dose influencing transcriptomic responses. Importantly, a high infectious dose of PDCoV infection was associated with greater inflammatory response and immune response. Furthermore, STAT1 and its downstream ISGs were shown to participate in the antiviral immune responses against PDCoV infection. Overall, our experiments underscore the necessity of incorporating dose-dependent parameters into PDCoV risk prediction for swine population.

## Data Availability

The authors confirm that the data supporting the findings of this study are available within the article and its Supplementary Materials.

## Supplementary Materials

The following supporting information can be downloaded at: https://www.mdpi.com/article/10.3390/ani15172536/s1, Figure S1. The mRNA levels of *IL6* (A), *ISG20* (B), *MX2* (C), *TNF-α* (D), *TRIM21* (E), *C3* (F), *CHI3L1* (G), *F3* (H), *MEGF9* (I), *SOD3* (G), and *TIMP2* (H) were compared with RT-qPCR. Gene expression levels, as measured by RT-qPCR, are plotted on the left axis, while RNA-seq expression levels in TPM units are plotted on the right axis. Ns: not significant; * *p* < 0.05; ** *p* < 0.01. Figure S2. (A–C) GO enrichment analysis was conducted on upregulated DEGs across different comparison groups. (D) Overlapping DEGs among different comparison groups and their associated GO terms. Table S1. Primer sequences for RT-qPCR. Table S2. Singaling pathways in L_PDCoV vs. Mock, H_PDCoV vs. Mock and H_PDCoV vs. L_PDCoV. Table S3. KEGG pathway enrichment analysis. Table S4. Genes related to “defense response to virus”.
