# Supplementary material for: Dose-Dependent Porcine Deltacoronavirus Infection Reveals Linkage Between Infectious Dose and Immune Response
Source: Animals (Basel). 2025 Aug 28;15(17):2536. doi: 10.3390/ani15172536 (PMC12427545; doi:10.3390/ani15172536)
Supplement: Supplementary file 1 [file animals-15-02536-s001.zip › Supplementary Materials.pdf]

Supplementary Materials

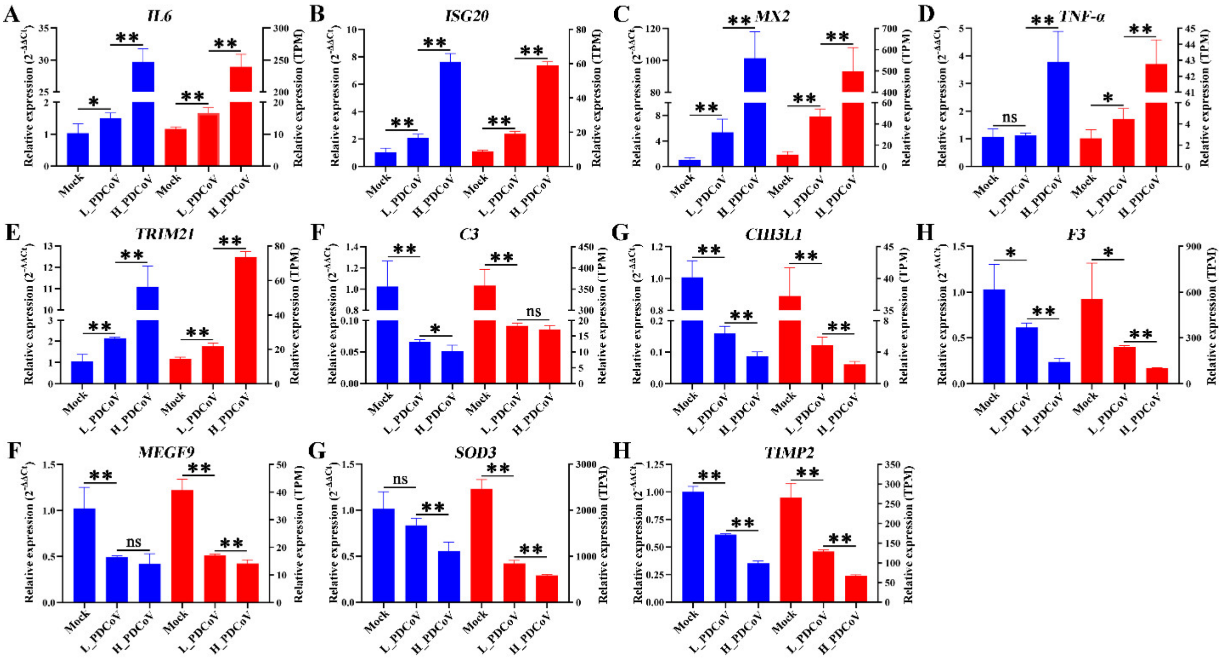

Figure S1. The mRNA levels of *IL6* (A), *ISG20* (B), *MX2* (C), *TNF-α* (D), *TRIM21* (E), *C3* (F), *CHI3L1* (G), *F3* (H), *MEGF9* (I), *SOD3* (G), and *TIMP2* (H) were compared with RT-qPCR. Gene expression levels, as measured by RT-qPCR, are plotted on the left axis, while RNA-seq expression levels in TPM units are plotted on the right axis. Ns not significant, * $p < 0.05$, ** $p < 0.01$.

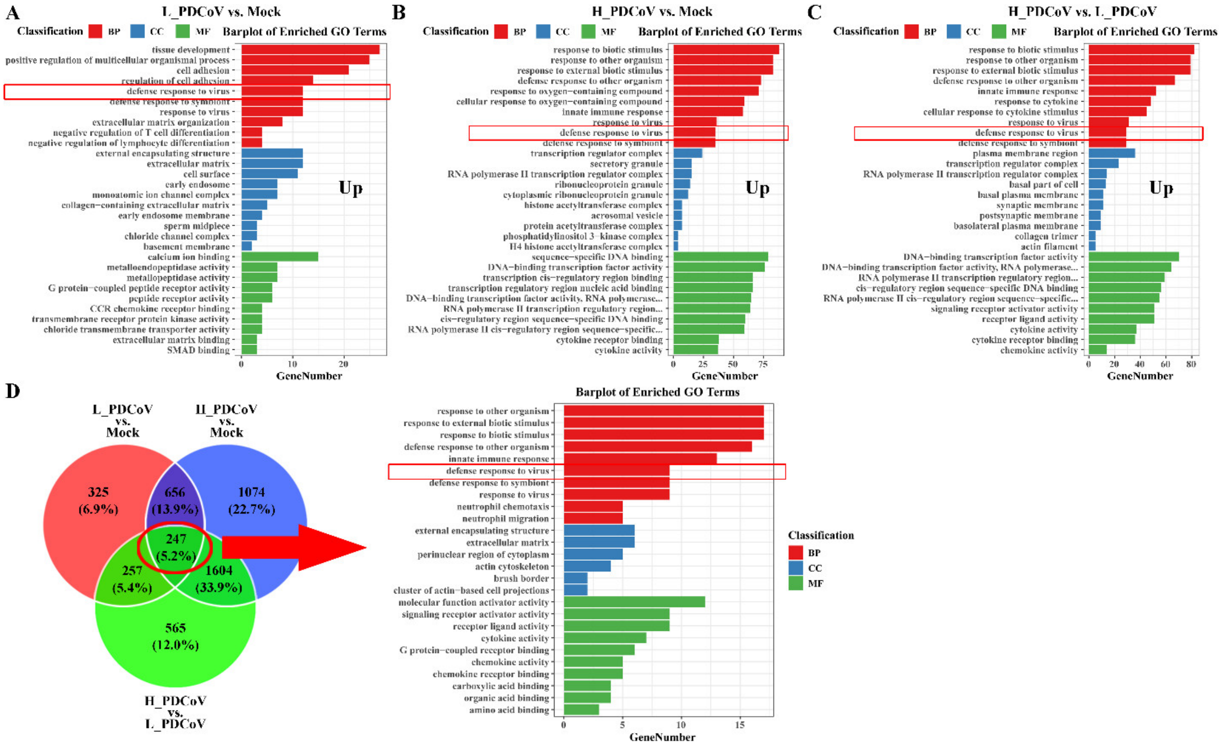

Figure S2. (A-C) GO enrichment analysis was conducted on upregulated DEGs across different comparison groups. **(D)** Overlapping DEGs among different comparison groups and their associated GO terms.
